# Incidence, Prognostic Factors and Survival for Hemangioblastoma of the Central Nervous System: Analysis Based on the Surveillance, Epidemiology, and End Results Database

**DOI:** 10.3389/fonc.2020.570103

**Published:** 2020-09-09

**Authors:** Xiangdong Yin, Hongzhou Duan, Zhiqiang Yi, Chunwei Li, Runchun Lu, Liang Li

**Affiliations:** Department of Neurosurgery, Peking University First Hospital, Beijing, China

**Keywords:** hemangioblastoma, SEER, incidence, treatment, survival, prognosis, nomogram

## Abstract

**Background:**

Hemangioblastomas are uncommon, benign neoplasms of the central nervous system (CNS). This study aims to evaluate the incidence, demographics, clinical characteristics, and prognosis of CNS hemangioblastomas using the data from the Surveillance, Epidemiology, and End Results (SEER) Program.

**Methods:**

Univariate and multivariate analyses using the Cox proportional hazards model were employed to identify prognostic factors of overall survival. The Kaplan-Meier method was utilized to evaluate overall survival distribution by treatment modality. A nomogram was further built to predict survival at 3 and 5 years.

**Results:**

The overall incidence rate of CNS hemangioblastomas was 0.141 per 100,000 person-years. Through univariate analysis and multivariate analyses, age between 60 and 79 years (HR = 3.697, *p* < 0.001), age greater than 80 years (HR = 12.318, *p* < 0.001), African American race (HR = 1.857, *p* = 0.003), multiple tumors (HR = 1.715, *p* < 0.001), and prior surgery (HR = 0.638, *p* = 0.013) were significantly associated with overall survival. Patients receiving surgery alone had better overall survival compared with patients receiving no treatment (*p* = 0.008) and patients receiving both surgery and radiotherapy (*p* = 0.002). The calibration plots demonstrated an excellent agreement between nomogram-predicted and actual survival.

**Conclusion:**

In conclusion, age, race, tumor location, number of tumors, and prior surgery are prognostic factors for survival. Surgery was the most common modality and was suggested as an effective and optimal treatment. The proposed nomogram can predict the prognosis of patients with CNS hemangioblastomas and help clinicians in making decisions.

## Introduction

Hemangioblastomas are uncommon, benign neoplasms (WHO grade I) of the central nervous system (CNS) that may appear either sporadically or as a part of von Hippel-Lindau disease, along with a variety of benign and malignant tumors (1). Hemangioblastomas account for approximately 2% of the intracranial neoplasms and 2–10% of the primary spinal cord neoplasms (2, 3). These highly vascularized tumors are most commonly located in the cerebellum (45%–50%), followed by the spinal cord (40–45%) and brain stem (5–10%) (4–6). CNS hemangioblastomas usually present with unpredictable growth (7, 8). Although CNS hemangioblastomas do not invade or metastasize, they can cause symptoms by tumor-related bleeding or compression of the adjacent structure.

Both surgery and radiotherapy have a role in the management of CNS hemangioblastomas (9). Most CNS hemangioblastomas can be safely resected, and gross total resection offers definitive therapy (9–12). Radiation therapy is also an option, especially for patients with residual, recurrent, or surgically inaccessible lesions (13, 14). The rarity of CNS hemangioblastomas has limited the studies regarding the epidemiological and clinical characteristics, and patient survival. Given this gap in the literature, we identified a representative cohort to evaluate the incidence, demographic or clinical characteristics, and prognosis of CNS hemangioblastomas. The Surveillance, Epidemiology, and End Results (SEER) database, which collects cancer statistics that cover nearly 28% of the United States population, was used to identify a relatively large population-based cohort of patients with CNS hemangioblastomas.

## Materials and Methods

### Data Collection

Data on patients with CNS hemangioblastomas were extracted from the SEER database for the year 2000 to 2016. All cases were identified using the ICD for Oncology (ICD-O-3) histology codes:9161/0 (acquired tufted hemangioblastoma); 9161/1 (hemangioblastoma); and 9161/3 (hemangioblastoma, malignant). All records were found in the following locations: C70.0 (cerebral meninges), C70.1 (spinal meninges), C70.9 (Meninges, NOS), C71.0 (cerebellum), C71.1 (frontal lobe), C71.2 (temporal lobe), C71.3 (parietal lobe), C71.4 (occipital lobe), C71.5 (ventricle), C71.6 (cerebellum, NOS), C71.7 (brain stem), C71.8 (overlapping lesion of brain), C71.9 (brain, NOS), C72.0 (spinal cord), C72.1 (cauda equina), C72.2 (olfactory nerve), C72.3 (optic nerve), C72.4 (acoustic nerve), C72.5 (cranial nerve, NOS), C72.8 (overlapping lesion of brain and CNS), C72.9 (nervous system, NOS), C75.1 (pituitary gland), C75.2 (craniopharyngeal duct) and C75.3 (pineal gland). Following the SEER database policies, both the incidence and case data were extracted with SEER^∗^Stat software.

Incidence rates of CNS hemangioblastomas were analyzed using SEER^∗^Stat with 95% confidence intervals (CI) and adjustment to the 2000 US Standard population. Both the frequency counts and percentages were depicted. Age-adjusted incidence rates were calculated as the number of CNS hemangioblastoma cases per 100,000 person-years. The incidence rates were also computed by age, gender, and race category, where a *p*-value for incidence rate ratios (IRRs) <0.05 was considered significant.

Age at diagnosis was classified into five groups: 0–19 years, 20–39 years, 40–59 years, 60–79 years, and over 80 years. Gender was divided into male and female. Marital status was dichotomized to Married (coded as “married, including common law”), and Unmarried (coded as “single, never married,” “divorced,” “separated,” “widowed,” or “unmarried or domestic partner”). Race was classified into four groups: Caucasian (coded as “White”), African American (coded as “Black”), Other (coded as “American Indian/Alaska Native” or “Asian or Pacific Islander”), and Unknown (coded as “Unknown”). The tumor size was classified as follows: 0–20 mm, 20–40 mm, and larger than 40 mm. The numbers of CNS hemangioblastomas were divided into solitary tumor and multiple tumors.

Tumor location was divided into five categories: cerebellum (coded as C71.6 Cerebellum, NOS), brain stem (coded as C71.7 Brain stem), spinal cord (coded as C70.1 Spinal meninges; C72.0 Spinal cord; C72.1 Cauda equina), cerebrum (coded as C71.0 Cerebrum; C71.1 Frontal lobe; C71.2 Temporal lobe; C71.3 Parietal lobe; C71.4 Occipital lobe), and other locations (coded as C72.3 Optic nerve; C72.4 Acoustic nerve; C75.1 Pituitary gland; C75.3 Pineal gland). Unclear or unknown locations (coded as C71.9 Brain, NOS; C72.9 Nervous system, NOS; C71.5 Ventricle NOS; C71.8 Overlapping lesion of brain; C72.8 Overlapping lesion of brain and CNS; C72.5 Cranial nerve, NOS) were excluded in the location analysis.

Treatment modality was divided into four groups: surgery alone, radiotherapy alone, both surgery and radiotherapy, and neither surgery nor radiotherapy. Unknown status of surgery or radiation therapy was not included in the analysis. The extent of tumor resection was classified as follows. Gross total resection was coded as “radical, total, gross resection of tumor, lesion or mass in brain (30) and gross total resection of lobe of brain (55).” Partial resection was coded as “tumor destruction, NOS (10); local excision of tumor, lesion, or mass excisional biopsy (20/21/22), and partial resection of lobe of brain (40).” Unknown surgery status, which was coded as “unknown if surgery performed (99) and Surgery, NOS (90),” was excluded. The outcome in this study was overall survival time. The date of death or censoring was obtained from the SEER database. Cases without data on survival time were not included in the survival analysis.

### Statistical Analysis

Statistical analyses were performed using IBM SPSS 23 (IBM SPSS, Inc., Chicago, IL, United States) and R version 3.6.0 (R Foundation for Statistical Computing, Vienna, Austria). For univariate analysis, the Cox proportional hazards model was utilized to evaluate the relationship between demographic/treatment variables and overall survival. All variables found to exhibit *p* < 0.10 in univariate analyses were included in multivariate analyses, which also employed the Cox proportional hazards model. Survival curves were depicted with the Kaplan-Meier method and compared using the log-rank test. Overall survival distributions by treatment modality were further evaluated with the Kaplan-Meier method. The nomogram was depicted based on the multivariate analysis (15) and by using the “rms” package in R version 3.6.0. The performance of the nomogram was assessed using concordance index (C-index) and calibration curve (16). All statistical tests were two-sided. A *p*-value <0.05 was considered statistically significant.

## Results

### Patient Characteristics

The incidence was estimated by age, gender, and race (Table 1). The overall incidence rate (IR) of CNS hemangioblastomas was 0.141 per 100,000 person-years (95% CI = 0.135–0.147). The incidence rate was relatively low in the younger patients of 0–19 years, with an age-adjusted incidence of 0.036 per 100,000 person-years (95% CI = 0.030–0.042). Hemangioblastomas were most frequent in patients of 60–79 years (0.246 per 100,000 person-years, 95% CI = 0.224–0.269). The incidence rates for females and males were 0.127 (95% CI = 0.119–0.135) and 0.158 (95% CI = 0.149–0.167) per 100,000 person-years respectively, representing an increased incidence rate ratio among male compared to female [Incidence rate ratio (IRR) = 1.246, 95% CI = 1.141–1.361, *p* < 0.001]. Incidence rate was the highest in Caucasians, with an adjusted incidence of 0.145 per 100,000 person-years (95% CI = 0.138–0.153). Compared with Caucasians, the incidence rates were lower in African Americans (IR = 0.121, 95% CI = 0.105–0.139; IRR = 0.833, 95% CI = 0.714–0.967, *p* < 0.05), American Indians/Alaska Natives (IR = 0.133, 95% CI = 0.089–0.195; IRR = 0.918, 95% CI = 0.608–1.346, *p* > 0.05), and Asians or Pacific Islanders (IR = 0.110, 95% CI = 0.093–0.128; IRR = 0.754, 95% CI = 0.636–0.889, *p* < 0.05).

**TABLE 1:** Age-adjusted incidence for CNS hemangioblastoma as a function of demographic variables.

Variable	Number of cases (%)	Incidence rate (per 100,000)	95% CI
**Age**	2,062	0.141	0.135–0.147
0–19	145	0.036	0.030–0.042
20–39	587	0.146	0.134–0.158
40–59	787	0.200	0.186–0.214
60–79	484	0.246	0.224–0.269
80+	59	0.125	0.095–0.162
**Gender**	2,062	0.141	0.135–0.147
Female	947	0.127	0.119–0.135
Male	1,115	0.158	0.149–0.167
**Race**	2,062	0.141	0.135–0.147
Caucasian	1,634	0.145	0.138–0.152
African American	203	0.121	0.105–0.139
American Indian/Alaska Native	29	0.133	0.089–0.195
Asian or Pacific Islander	161	0.110	0.093–0.128
Unknown	35	NS^1^	NS^1^

*^1^Not significant.*

There were 2,062 cases of CNS hemangioblastomas identified in the SEER-18Regs database. The mean age at diagnosis of the patients with CNS hemangioblastomas was 47.0 ± 17 years. The majority of patients were males (54.1%) and Caucasians (79.2%). Approximately 52.5% of the patients were married at the age of diagnosis. The majority of CNS hemangioblastomas were located in the cerebellum (62.2%), followed by spinal cord (14.3%), cerebrum (6.2%), and brainstem (5.1%). Overall, 87% of all the patients received medical interventions. Surgical treatment was performed in 87.4% of the patients. Radiation therapy was administered in 5.3% of the patients.

### Survival Analysis

**FIGURE 1:**
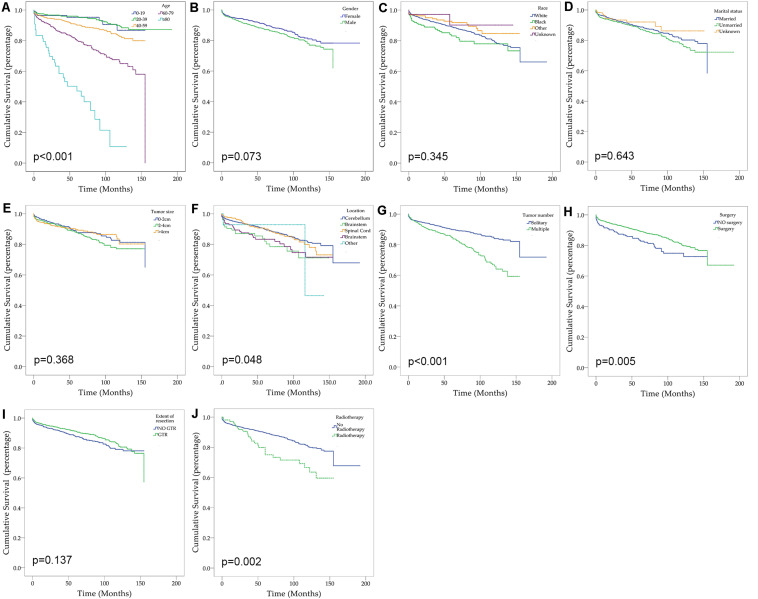
Kaplan–Meier curves for patients with CNS hemangioblastomas by different variates. **(A)** Age; **(B)** Gender; **(C)** Race; **(D)** Marital status; **(E)** Tumor size; **(F)** Tumor location; **(G)** Tumor number; **(H)** Prior surgery; **(I)** Extent of resection; **(J)** Prior radiotherapy.

After excluding three cases without survival data, we finally identified 2,059 cases for the following analyses (Figure 1 and Table 2). The overall 1-, 3-, and 5-year survival estimates were 95.30% (95% CI = 94.20-96.10%), 92.00% (95% CI = 90.60-93.20%), and 88.80% (95% CI = 87.20–90.30%) respectively. According to the univariate analysis, age 60–79 years [vs age 0–19 years; hazard ratio (HR) = 3.814, 95% CI = 1.929–7.539, *p* < 0.001], age greater than 80 years (vs age 0–19 years; HR = 13.748, 95% CI = 6.530–28.945, *p* < 0.001), male (vs female, HR = 1.250, 95% CI = 0.979–1.596, *p* = 0.074), African Americans (vs Caucasians; HR = 1.401, 95% CI = 0.971–2.022, *p* = 0.071), location of brainstem (vs cerebellum, HR = 1.826, 95% CI = 1.144–2.913, *p* = 0.012), location of cerebrum (vs cerebellum, HR = 1.614, 95% CI = 1.064–2.447, *p* = 0.024), multiple tumors (vs solitary tumor; HR = 2.013, 95% CI = 1.574–2.574, *p* < 0.001) and prior radiotherapy (vs no radiotherapy; HR = 1.888, 95% CI = 1.259–2.831, *p* = 0.002) were significantly associated with decreased overall survival. The prior surgery (vs no surgery, HR = 0.644, 95% CI = 0.471–0.880, *p* = 0.006) was associated with better overall survival. Marital status, tumor size and extent of tumor resection were not significantly associated with survival (*p* > 0.1).

**TABLE 2:** Univariate and multivariate analyses of survival in patients with CNS hemangioblastoma.

Variable	Patients	5-year survival rate (%)	Univariate survival		Multivariate survival	
			HR (95% CI)	*p*	HR (95% CI)	*p*
**Age**	2,059	88.8				
≤19	145	95.3	1 [Reference]		1 [Reference]	
20–39	587	96.0	0.940 (0.455–1.945)	0.868	0.902 (0.428–1.901)	0.787
40–59	787	90.2	1.688 (0.849–3.352)	0.135	1.411 (0.696–2.860)	0.340
60–79	483	79.9	3.814 (1.929–7.539)	<0.001	3.697 (1.837–7.442)	<0.001
≥80	57	47.1	13.748 (6.530–28.945)	<0.001	12.318 (5.625–26.974)	<0.001
**Gender**	2,059	88.8				
Female	946	90.4	1 [Reference]		1 [Reference]	
Male	1,113	87.2	1.250 (0.979–1.596)	0.074	1.278 (0.979–1.668)	0.072
**Race**	2,059	88.8				
Caucasian	1,632	88.9	1 [Reference]	0.084	1 [Reference]	
African American	203	83.1	1.401 (0.971–2.022)	0.071	1.857 (1.237–2.786)	0.003
Other	189	91.8	0.712 (0.440–1.152)	0.166	0.937 (0.560–1.569)	0.805
Unknown	34	90.2	0.516 (0.128–2.078)	0.352	0.436 (0.061–3.130)	0.436
**Marital status**	2,059	88.8				
Married	1,080	88.9	1 [Reference]			
Unmarried	855	87.8	1.202 (0.942–1.535)	0.140		
Unknown	124	92.1	0.732 (0.385–1.391)	0.340		
**Tumor size (cm)**	1,347	88.2				
≤2	402	87.7	1 [Reference]			
2–4	595	87.6	1.250 (0.874–1.787)	0.211		
≥4	350	89.8	1.020 (0.670–1.552)	0.926		
**Tumor location**	1,842	88.9				
Cerebellum	1,280	89.8	1 [Reference]		1 [Reference]	
Brainstem	105	82.0	1.826 (1.144–2.913)	0.012	2.075 (1.289–3.342)	0.003
Spinal cord	295	90.0	1.078 (0.749–1.551)	0.687	1.092 (0.755–1.581)	0.640
Cerebrum	147	83.3	1.614 (1.064–2.447)	0.024	1.520 (1.012–2.343)	0.044
Other	14	92.9	1.223 (0.303–4.937)	0.777	1.616 (0.397–6.581)	0.503
**Tumor number**	2,059	88.8				
Solitary	1,579	90.0	1 [Reference]		1 [Reference]	
Multiple	480	84.8	2.013 (1.574–2.574)	<0.001	1.715 (1.307–2.251)	<0.001
**Surgery**	2,053	88.7				
No	251	83.4	1 [Reference]		1 [Reference]	
Yes	1,802	89.5	0.644 (0.471–0.880)	0.006	0.638 (0.448–0.908)	0.013
**GTR**	1,770	89.5				
No	805	87.5	1 [Reference]			
Yes	965	91.1	0.816 (0.624–1.068)	0.139		
**Radiotherapy**	2,052	88.8				
No	1,942	89.6	1 [Reference]		1 [Reference]	
Yes	110	75.0	1.888 (1.259–2.831)	0.002	0.928 (0.574–1.500)	0.760

**FIGURE 2:**
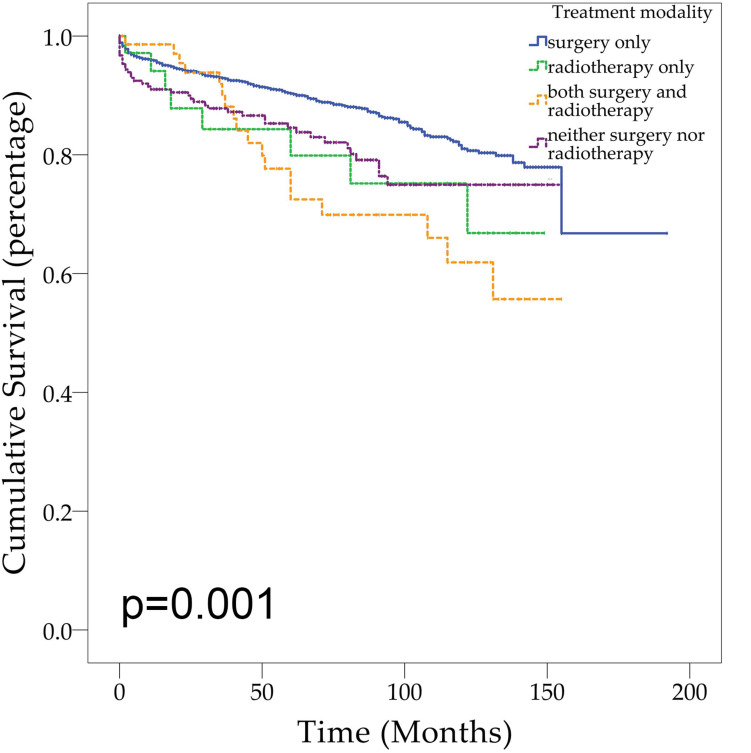
Kaplan–Meier curves for patients with CNS hemangioblastomas by treatment modality.

Demographic or clinical variables (age, gender, race, tumor location, number of tumors, prior surgery, and prior radiation) with *p* < 0.1 from the univariate analysis were included in the multivariate analysis (Table 2). On multivariate Cox regression among patients with CNS hemangioblastomas, the age groups of 60–79 years (HR = 3.697, 95% CI = 1.837–7.442, *p* < 0.001) and greater than 80 years (HR = 12.318, 95% CI = 5.625–26.976, *p* < 0.001), remained associated with an increased hazards ratio for death compared with patients of 0–19 years. African American race also remained associated with an increased hazards ratio for death compared with the Caucasian race (HR = 1.857, 95% CI = 1.237–2.786, *p* = 0.003). Patients with multiple tumors had significantly worse overall survival than those with solitary hemangioblastoma (HR = 1.715, 95% CI = 1.307–2.251, *p* < 0.001). Prior surgery was associated with improved overall survival (HR = 0.638, 95% CI = 0.448–0.908, *p* = 0.013). On the multivariate analysis, gender (*p* = 0.072) and prior radiotherapy (*p* = 0.760) were no longer significant predictors of overall survival. Among all the patients, 84.1% received surgery alone, 1.9% received radiotherapy alone, 3.6% received both surgery and radiotherapy, and 10.4% received neither surgery nor radiotherapy (Figure 2 and Table 3). The percentages of cases surviving 5 years by treatment modality were: 90.3% for surgery alone, 79.9% for radiotherapy alone, 72.5% for both surgery and radiotherapy, and 84.6% for neither surgery nor radiotherapy. Distribution of overall survival among patients who received surgery alone was significantly different from patients who received both surgery and radiotherapy (*p* = 0.002) and from patients who received neither surgery nor radiotherapy (*p* = 0.008). Other comparisons of distributions of treatment modality were not significantly different.

**TABLE 3:** Distribution of treatment modality and survival for CNS hemangioblastoma.

Treatment	Cases (%)	75% survival time	3-year survival (%)	5-year survival (%)	HR	*p*
**Surgery alone**	1722 (84.1%)	155	92.9	90.3	1 [Reference]	
**Radiotherapy alone**	38 (1.9%)	122	84.3	79.9	1.764	0.116
**Both surgery and radiotherapy**	74 (3.6%)	60	90.0	72.5	2.148	0.002
**Neither surgery nor radiotherapy**	214 (10.4%)	94	87.8	84.6	1.588	0.008

### Nomogram

**FIGURE 3:**
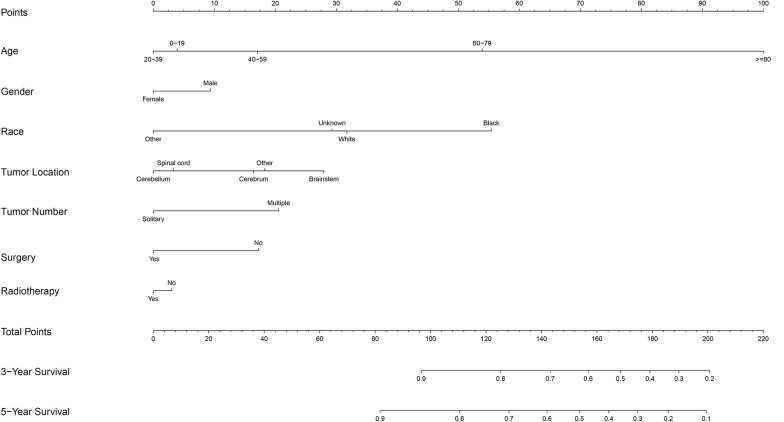
Nomograms predicting 3- and 5-year survival of patients with CNS hemangioblastoma.

**FIGURE 4:**
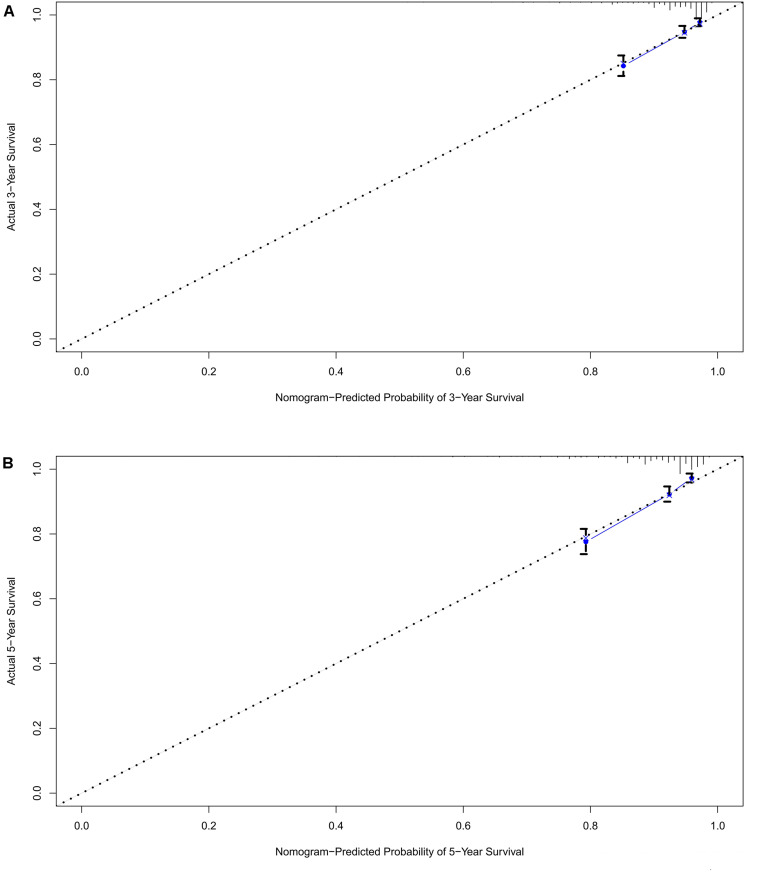
Calibration plots of the nomogram for OS prediction at 3-year **(A)** and 5-year **(B)**.

A prognostic nomogram was analyzed using the demographic and clinical variables with *p* < 0.1 from the univariate analysis. The nomogram for survival at 3 or 5 years is shown in Figure 3. The C-index for survival prediction was 0.73 (95% CI = 0.71–0.75). The calibration plots for the probability of survival at 3 and 5 years demonstrated an excellent agreement between the predictive nomogram and actual observation (Figure 4). We also created an online nomogram prognosis calculator to assist clinicians and researchers. The online nomogram can be accessed at https://neurosurgxd.shinyapps.io/cns-hemangioblastoma/.

## Discussion

To our knowledge, this study of the SEER database represents the largest published cohort of patients with CNS hemangioblastomas at present. This study analyzed the incidence, demographic and clinical characteristics, and prognosis of CNS hemangioblastomas, which may be more generalizable than previously published literature with relatively small case series (1, 7, 10–12, 17–19). Our analysis of incidence indicates that CNS hemangioblastomas are relatively rare neoplasms, with an overall incidence of 0.141 per 100,000 person-years and highest incidence among Caucasian males between age 65–69. Based on the univariate analysis and multivariate analyses, age at diagnosis, race, tumor location, number of tumors, and prior surgery are significantly associated with overall survival. However, gender, marital status, tumor size, the extent of tumor resection, and prior radiotherapy did not demonstrate significant associations with overall survival.

Our study demonstrated decreased overall survival among patients with age greater than 60 compared with younger patients, which was most likely caused by the shortened life expectancy in aged patients. Comorbid conditions that are associated with age may play a role in the mortality of aged patients. Furthermore, worse access to high-quality neuro-oncologic care in aged patients may also contribute to the survival differences. We also noticed a predominance of CNS hemangioblastomas in males, with a male to female ratio of 1.25:1. According to the analysis of incidence, Caucasians had significantly greater odds of being diagnosed with CNS hemangioblastomas compared with other races. Similar incidence patterns have been observed previously in multiple different neoplasms (20). It was suggested that gender or race specific oncological mutations and environmental and lifestyle factors may be associated with the differences in incidence (21, 22). Despite the highest incidence among Caucasians, African American patients with CNS hemangioblastomas had significantly worse overall survival than Caucasian patients. This finding may be explained by many reasons. First, the racial categorization of the SEER database fails to sufficiently reflect the genetic differences and complexity of patients’ racial and ethnic backgrounds. Secondly, a portion of the survival differences may be the result of the comparatively fewer cases among African American patients. In addition, many factors, which vary by race or ethnicity, are presumably associated with survival after diagnosis of cancer of other sites, including body mass index, smoking status, religious attendance, and biological differences. However, the roles of these factors have not been adequately studied. More importantly, it has been suggested by numerous studies that African Americans are less likely to receive optimal care, especially high-quality neuro-oncologic care, than Caucasians and are also presented with greater disease severity. Such inequalities may substantially contribute to the survival differences. The objective of public health and future research should be finding remedial measures for the health inequities rather than exploring more variations between different racial groups.

Nearly 34% of CNS hemangioblastomas appear in the context of VHL disease and the remainder appear sporadically (4). The relationship of CNS hemangioblastoma with VHL disease could not be obtained from the SEER database, but approximately 23.3% of the patients in this cohort have multiple hemangioblastomas, which were usually associated with VHL disease (23). Patients with multiple lesions had significantly worse survival than patients with a solitary tumor. Multiple hemangioblastomas usually appear in more than 90% of patients with VHL disease (23), which may increase the risks of morbidity and mortality due to the involvement of viscera (18). Visceral lesions potentially add complexity to the treatment of these patients. Furthermore, the higher risks and difficulties in resecting multiple lesions may also contribute to the worse survival in these patients.

In this study, CNS hemangioblastomas are most frequently located in the cerebellum (62.2%), which was consistent with previously published studies (18). We found that overall survival for patients with CNS hemangioblastomas varied significantly by tumor location. Decreased survival time was significant in patients with hemangioblastomas located in the brainstem or other locations (cranial nerves or glands). Surgical treatment of brainstem tumors has always been considered to be a challenge for neurosurgeons. The complex anatomical structures and crucial neural structures of the brainstem create a higher risk for morbidity and mortality. However, with advances in microsurgical techniques, surgery for brainstem hemangioblastoma has become safe and feasible (24). Hemangioblastomas arising from cranial nerves or glands are extremely rare, with only 14 cases from the SEER database. Currently, little is known about the features of those lesions, and more studies are required to explore their characteristics.

Within this SEER cohort, 87.4% of the patients with CNS hemangioblastomas underwent surgical treatment. On both univariate and multivariate analyses, prior surgery was a significant predictor for better overall survival. This finding is consistent with the current consensus that surgery offers definitive therapy for patients with CNS hemangioblastomas. However, the optimal timing of surgical intervention remains debated due to the unpredictable growth or progression of hemangioblastomas (25). Currently, the consensus among most neurosurgeons is that surgical treatment is reserved for symptomatic lesions (7). The role of radiographic progression in treatment decision-making is still controversial (8, 26). Notably, gross total resection in this analysis did not demonstrate significant associations with better survival, contrary to the pervasive notion that gross total resection is a therapeutic approach to cure CNS hemangioblastomas with improved outcomes. The lack of a significantly better survival among patients who received gross total resection may be related to the low rate of gross total resection and the controversial indications for surgery.

Despite the important role of surgery in the management of CNS hemangioblastomas, radiotherapy has been investigated as an alternative treatment strategy. Recent studies have shown that radiotherapy is a viable treatment option with acceptable local control and survival rates (1, 14). Approximately 5.3% of the patients received radiotherapy in this analysis. Surprisingly, based on the univariate analysis, patients who underwent radiotherapy had significantly worse overall survival than patients without radiotherapy. On multivariate analysis which included other qualifying factors, prior radiotherapy was no longer significantly associated with overall survival. One of the explanations for the absence of a survival advantage among patients treated with radiotherapy in this cohort is likely attributable to the relatively small sample size analyzed. Moreover, radiotherapy is typically reserved for patients with non-resectable, residual, and recurrent tumors, which may cause selection bias for this effect.

Based on the overall survival stratified by treatment modality, this analysis shows that patients receiving surgery alone had better overall survival compared with patients receiving no treatment and patients receiving both surgery and radiotherapy. There was no significant difference in overall survival between patients receiving surgery alone and patients receiving radiotherapy alone. To date, both surgery and radiotherapy have roles in the management of CNS hemangioblastomas. Early studies have suggested that surgical resection of hemangioblastomas, which contributes to the timely relief of regional pressure, remains the optimal option (10–12, 19). Gross total resection is curative (9). On the basis of this analysis and other clinical studies, it is advisable to suggest that surgery still represents an effective and optimal management for CNS hemangioblastomas.

Nomograms have been used for predicting prognosis in many neoplasms and have shown to be excellent in terms of predictive accuracy for survival (27, 28). In our study, we established a nomogram based on a large cohort from the SEER database for the first time. Seven variables were included in the nomogram: age, gender, race, location, the number of tumors, the prior surgery, and prior radiotherapy. Supported by the C-index and the calibration curves, our nomogram showed good predictive performance and could be used as a personalized predictive tool for prognosis in patients with CNS hemangioblastomas. However, future work with more detailed information and external samples are needed to improve its performance.

Several limitations should be considered in our study. This retrospective study of SEER data is not randomized and consequently may be confounded by many factors. Although the SEER database provides a large amount of valuable data, some important information is not available. For instance, we cannot differentiate between sporadic hemangioblastomas and VHL-related hemangioblastomas within the SEER database, which would have been important in the survival analysis (6, 29). In addition, other clinical characteristics including neurological symptoms, preoperative neurological status, and postoperative morbidity were not accessible, which were reported to be associated with patient prognosis (17, 30). Furthermore, detailed information regarding surgery including surgical approach, utilization of preoperative embolization, or intraoperative monitoring was not presented. These factors may influence patient outcomes and prognosis. Similarly, detailed data on radiotherapy including dosage, fields or fractionation, was not available. And the role of radiotherapy and surgery may not be fully investigated due to sample size, selection bias and natural history of CNS hemangioblastoma. Another limitation of this study is the presence of selection bias. Therefore, the results should be interpreted within the context of possible selection bias.

This population-based study of the largest series of patients with CNS hemangioblastomas from the SEER database demonstrates that hemangioblastomas are relatively rare neoplasms most commonly arising from the cerebellum. Demographically, CNS hemangioblastomas were more common in males, the aged, and Caucasian patients. Clinical factors including age at diagnosis, race, tumor location, number of tumors, and prior surgery were significantly associated with overall survival. Gender, marital status, tumor size, extent of tumor resection, and prior radiotherapy did not demonstrate significant relationships with overall survival. Surgery was the most common modality and was suggested as an effective and optimal treatment according to our analysis and other studies. Other important factors, which were not included in the SEER database, warrant further investigation. The proposed nomogram can predict the prognosis of patients with CNS hemangioblastomas and can assist clinicians in making decisions on an individual basis.

## Data Availability Statement

The raw data supporting the conclusions of this article will be made available by the authors, without undue reservation.

## Author Contributions

XY and LL designed the research, conducted the data analysis, interpreted the data, and wrote the main manuscript. HD, ZY, RL, and CL designed the research, supervised the data analysis, interpreted the data, and critically revised the article. All other co-authors helped to interpret the data and critically reviewed the article. All authors approved the final article for submission.

## Conflict of Interest

The authors declare that the research was conducted in the absence of any commercial or financial relationships that could be construed as a potential conflict of interest.

## References

[1] Kanno H Kobayashi N Nakanowatari S. Pathological and clinical features and management of central nervous system hemangioblastomas in von Hippel-Lindau disease. *J Kidney Cancer VHL.* (2014) 1:46–55. 10.15586/jkcvhl.2014.12 28326249 PMC5345529

[2] Rachinger J Buslei R Prell J Strauss C. Solid haemangioblastomas of the CNS: a review of 17 consecutive cases. *Neurosurg Rev.* (2009) 32:37–47. 10.1007/s10143-008-0166-0 18810515

[3] Bründl E Schödel P Ullrich OW Brawanski A Schebesch KM. Surgical resection of sporadic and hereditary hemangioblastoma: our 10-year experience and a literature review. *Surg Neurol Int.* (2014) 5:138. 10.4103/2152-7806.141469 25317353 PMC4192902

[4] Lonser RR Glenn GM Walther M Chew EY Libutti SK Linehan WM von Hippel-Lindau disease. *Lancet.* (2003) 361:2059–67. 10.1016/s0140-6736(03)13643-4 12814730

[5] Vortmeyer AO Falke EA Gläsker S Li J Oldfield EH. Nervous system involvement in von Hippel-Lindau disease: pathology and mechanisms. *Acta Neuropathol.* (2013) 125:333–50. 10.1007/s00401-013-1091-z 23400300

[6] Yousef A Rutkowski MJ Yalcin CE Eren OC Caliskan I Tihan T. Sporadic and Von-Hippel Lindau disease-associated spinal hemangioblastomas: institutional experience on their similarities and differences. *J Neurooncol.* (2019) 143:547–52. 10.1007/s11060-019-03189-w 31089924

[7] Lonser RR Butman JA Huntoon K Asthagiri AR Wu T Bakhtian KD Prospective natural history study of central nervous system hemangioblastomas in von Hippel-Lindau disease. *J Neurosurg.* (2014) 120:1055–62. 10.3171/2014.1.Jns131431 24579662 PMC4762041

[8] Ammerman JM Lonser RR Dambrosia J Butman JA Oldfield EH. Long-term natural history of hemangioblastomas in patients with von Hippel-Lindau disease: implications for treatment. *J Neurosurg.* (2006) 105:248–55. 10.3171/jns.2006.105.2.248 17219830

[9] Dornbos D III Kim HJ Butman JA Lonser RR. Review of the neurological implications of von Hippel-Lindau disease. *JAMA Neurol.* (2018) 75:620–7. 10.1001/jamaneurol.2017.4469 29379961

[10] Jagannathan J Lonser RR Smith R DeVroom HL Oldfield EH. Surgical management of cerebellar hemangioblastomas in patients with von Hippel-Lindau disease. *J Neurosurg.* (2008) 108:210–22. 10.3171/jns/2008/108/2/0210 18240914

[11] Lonser RR Weil RJ Wanebo JE DeVroom HL Oldfield EH. Surgical management of spinal cord hemangioblastomas in patients with von Hippel-Lindau disease. *J Neurosurg.* (2003) 98:106–16. 10.3171/jns.2003.98.1.0106 12546358

[12] Weil RJ Lonser RR DeVroom HL Wanebo JE Oldfield EH. Surgical management of brainstem hemangioblastomas in patients with von Hippel-Lindau disease. *J Neurosurg.* (2003) 98:95–105. 10.3171/jns.2003.98.1.0095 12546357

[13] Bridges KJ Jaboin JJ Kubicky CD Than KD. Stereotactic radiosurgery versus surgical resection for spinal hemangioblastoma: a systematic review. *Clin Neurol Neurosurg.* (2017) 154:59–66. 10.1016/j.clineuro.2017.01.012 28129633

[14] Pan J Jabarkheel R Huang Y Ho A Chang SD. Stereotactic radiosurgery for central nervous system hemangioblastoma: systematic review and meta-analysis. *J Neurooncol.* (2018) 137:11–22. 10.1007/s11060-017-2697-0 29204841

[15] Harrell FE Jr. Lee KL Mark DB. Multivariable prognostic models: issues in developing models, evaluating assumptions and adequacy, and measuring and reducing errors. *Stat Med.* (1996) 15:361–87. 10.1002/(sici)1097-0258(19960229)15:43.0.Co;2-4 8668867

[16] Pencina MJ D’Agostino RB. Overall C as a measure of discrimination in survival analysis: model specific population value and confidence interval estimation. *Stat Med.* (2004) 23:2109–23. 10.1002/sim.1802 15211606

[17] Fukuda M Takao T Hiraishi T Yoshimura J Yajima N Saito A Clinical factors predicting outcomes after surgical resection for sporadic cerebellar hemangioblastomas. *World Neurosurg.* (2014) 82:815–21. 10.1016/j.wneu.2014.06.018 24937595

[18] Kuharic M Jankovic D Splavski B Boop FA Arnautovic KI. Hemangioblastomas of the posterior cranial fossa in adults: demographics, clinical, morphologic, pathologic, surgical features, and outcomes. A systematic review. *World Neurosurg.* (2018) 110:e1049–62. 10.1016/j.wneu.2017.11.173 29229339

[19] Ordookhanian C Kaloostian PE Ghostine SS Spiess PE Etame AB. Management strategies and outcomes for VHL-related craniospinal hemangioblastomas. *J Kidney Cancer VHL.* (2017) 4:37–44. 10.15586/jkcvhl.2017.90 28868236 PMC5573741

[20] Mountain JL Risch N. Assessing genetic contributions to phenotypic differences among ‘racial’ and ‘ethnic’ groups. *Nat Genet.* (2004) 36(11 Suppl):S48–53. 10.1038/ng1456 15508003

[21] Burchard EG Ziv E Coyle N Gomez SL Tang H Karter AJ The importance of race and ethnic background in biomedical research and clinical practice. *N Engl J Med.* (2003) 348:1170–5. 10.1056/NEJMsb025007 12646676

[22] Kirsch-Volders M Bonassi S Herceg Z Hirvonen A Möller L Phillips DH. Gender-related differences in response to mutagens and carcinogens. *Mutagenesis.* (2010) 25:213–21. 10.1093/mutage/geq008 20194421

[23] Butman JA Linehan WM Lonser RR. Neurologic manifestations of von Hippel-Lindau disease. *JAMA.* (2008) 300:1334–42. 10.1001/jama.300.11.1334 18799446 PMC3487164

[24] Yin X Li C Li L Duan H. Safety and efficacy of surgical treatment for brainstem hemangioblastoma: a meta-analysis. *Neurosurg Rev.* (2020): 10.1007/s10143-020-01305-3. [Epub ahead of print]. 32356022 PMC8035120

[25] Harati A Satopää J Mahler L Billon-Grand R Elsharkawy A Niemelä M Early microsurgical treatment for spinal hemangioblastomas improves outcome in patients with von Hippel-Lindau disease. *Surg Neurol Int.* (2012) 3:6. 10.4103/2152-7806.92170 22347675 PMC3279991

[26] Feletti A Anglani M Scarpa B Schiavi F Boaretto F Zovato S Von Hippel-Lindau disease: an evaluation of natural history and functional disability. *Neuro Oncol.* (2016) 18:1011–20. 10.1093/neuonc/nov313 26763786 PMC4896541

[27] Touijer K Scardino PT. Nomograms for staging, prognosis, and predicting treatment outcomes. *Cancer.* (2009) 115(13 Suppl):3107–11. 10.1002/cncr.24352 19544538

[28] Wang Y Li J Xia Y Gong R Wang K Yan Z Prognostic nomogram for intrahepatic cholangiocarcinoma after partial hepatectomy. *J Clin Oncol.* (2013) 31:1188–95. 10.1200/jco.2012.41.5984 23358969

[29] Bamps S Calenbergh FV Vleeschouwer SD Loon JV Sciot R Legius E What the neurosurgeon should know about hemangioblastoma, both sporadic and in Von Hippel-Lindau disease: a literature review. *Surg Neurol Int.* (2013) 4:145. 10.4103/2152-7806.121110 24340227 PMC3841920

[30] Niu L Zhang Y Li Q Dai J Yin H Duan L The analysis of correlative factors affecting long-term outcomes in patients with solid cerebellar Hemangioblastomas. *Clin Neurol Neurosurg.* (2016) 150:59–66. 10.1016/j.clineuro.2016.08.028 27588372

